# Supplementary material for: Hippocampal astrocytes modulate anxiety-like behavior
Source: Nat Commun. 2022 Nov 7;13:6536. doi: 10.1038/s41467-022-34201-z (PMC9640657; doi:10.1038/s41467-022-34201-z)
Supplement: Supplementary file 1 — Supplementary Information [file 41467_2022_34201_MOESM1_ESM.pdf]

Supplementary Information

Hippocampal astrocytes modulate anxiety-like behavior

Woo-Hyun Cho[1,#,*], Kyungchul Noh[1,#], Byung Hun Lee[2], Ellane Barcelon[1], Sang Beom Jun[3,4,5], Hye Yoon Park[2,6], Sung Joong Lee[1,*]

[1]Department of Physiology and Neuroscience, Dental Research Institute, Seoul National University School of Dentistry, Seoul, 08826, Republic of Korea

[2]Department of Physics and Astronomy, Seoul National University, Seoul, 08826, Republic of Korea

[3]Department of Electronic and Electrical Engineering, Ewha Womans University, Seoul, 03760, Republic of Korea

[4]Graduate Program in Smart Factory, Ewha Womans University, Seoul, 03760, Republic of Korea

[5]Department of Brain & Cognitive Sciences, Ewha Womans University, Seoul, 03760, Republic of Korea

[6]Department of Electrical and Computer Engineering, University of Minnesota, Minneapolis, MN 55455, USA

[*]Corresponding author

[#] These authors contributed equally to this work.

Corresponding authors:
Sung Joong Lee, Ph.D.
Department of Physiology and Neuroscience, Dental Research Institute, Seoul National University School of Dentistry, 1 Gwanak-ro, Gwanak-gu, Seoul 08826, Republic of Korea
Phone: +82-2-880-2309; E-mail address: sjlee87@snu.ac.kr

Woo-Hyun Cho, Ph.D.
Department of Physiology and Neuroscience, Dental Research Institute, Seoul National University School of Dentistry, 1 Gwanak-ro, Gwanak-gu, Seoul 08826, Republic of Korea
E-mail address: woohyuncho15@snu.ac.kr

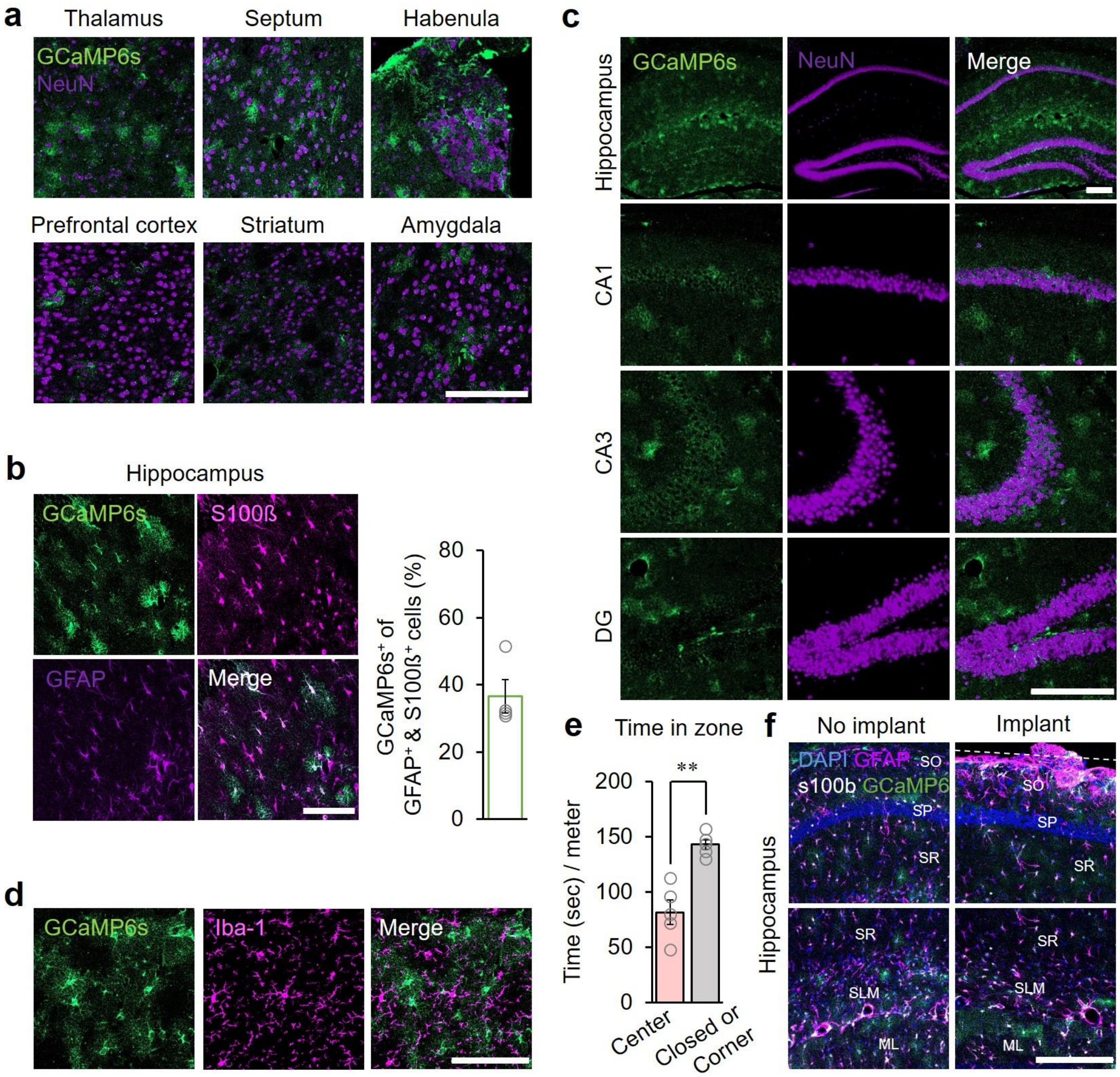

Supplementary figure 1. Astrocyte-specific GCaMP6s expression in hGFAP-GCaMP6s mice. **a,** GCaMP6s signal (green) did not merge with neurons ($NeuN^+$, purple) in various brain sub-regions. Scale bar, 200 μm. Representative data from three independent experiments. **b,** Left, Representative confocal image showing GCaMP6s (green) colocalization in astrocytes ($S100\beta^+$, pink and $GFAP^+$, purple) in hippocampus. Scale bar, 100 μm. Right, Percentage of GCaMP6s-expressing astrocytes in heterozygote hFGAP-GCaMP6s hippocampus (n = 4 mice). **c,** GCaMP6s

signal (green) did not merge with neurons ($NeuN^+$, purple) in hippocampal subregions, CA1, CA3, and DG, Scale bar, 200 μm. Representative data from three independent experiments. **d,** Representative confocal images showing GCaMP6s (green) colocalization in microglia ($Iba\text{-}1^+$, purple) in other brain areas. Scale bar, 100 μm. Representative data from three independent experiments. **e,** The time spent per meter when mice were in center area or closed or corner area (n = 5 mice). Two-tailed Mann-Whitney *U*-test ($p = 0.009$). **f,** Representative confocal image of hippocampal astrocyte morphology ($S100\beta^+$, white and $GFAP^+$, pink) with or without implant. Green, $GCaMP6s^+$; Blue, $DAPI^+$; SO, stratum oriens; SP, stratum pyramidale; SR, stratum radiatum; SLM, stratum lacunosum moleculare; ML, moleculare layer. Scale bar, 200μm. Representative data from three independent experiments. Bar graphs are represented as mean ± SEM. **, $p < 0.01$. See Supplementary Data for detailed statistics. Source data are provided as a Source Data file.

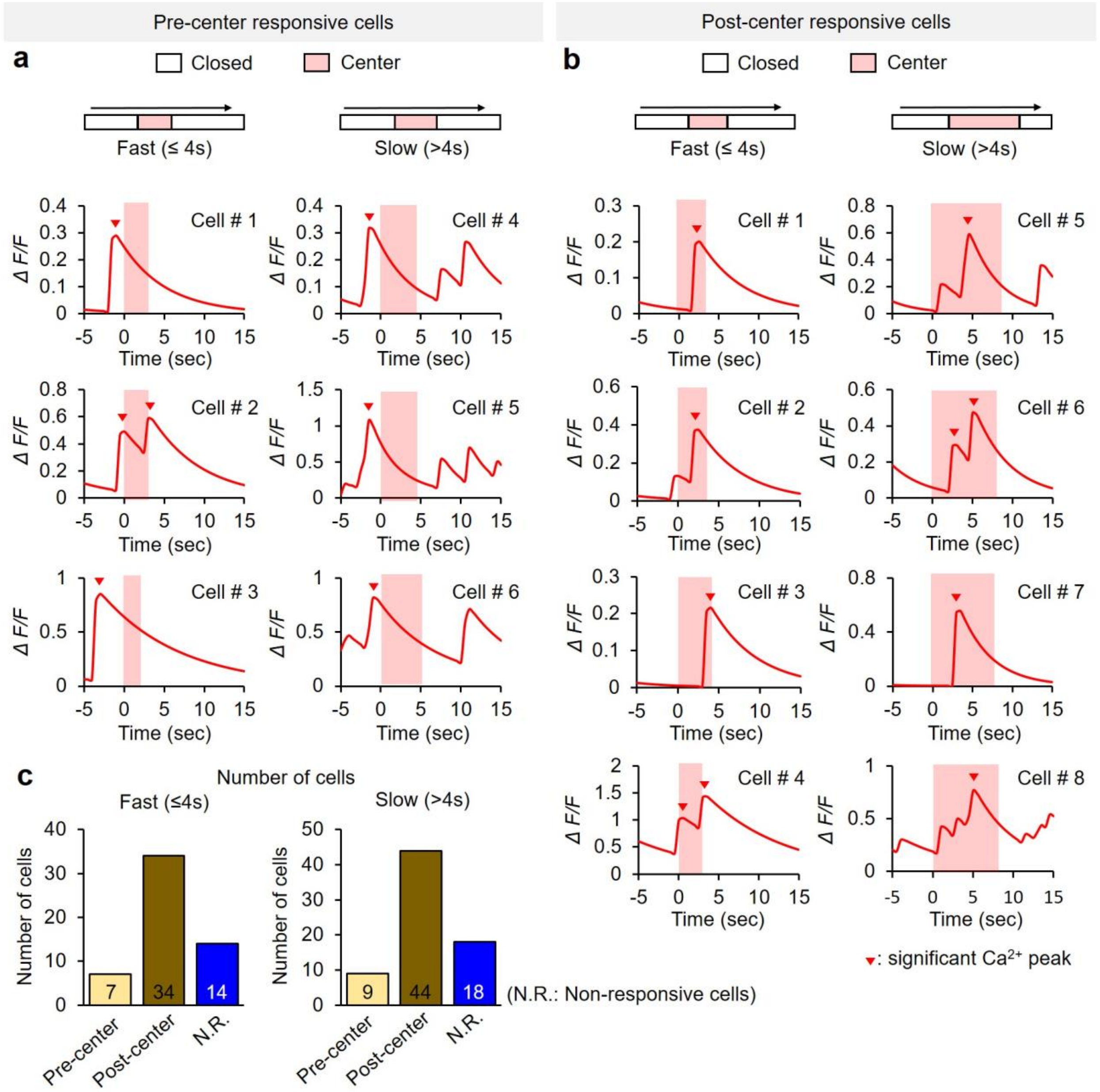

Supplementary figure 2. Ca^{2+} response in hippocampal astrocytes while mice exploring EPM in VR. Representative traces of astrocyte GCaMP6s signals when mice were entering the center area for 20 sec, sorted by the time mice spent passing the center (fast ≤ 4 sec, slow > 4 sec). **a,** Representative $Ca2^{+}$ traces that peaked 0.5 to 3.5 sec before mice entered the center area (Pre-center responsive cells). Pink bar and white background in each graph indicate center and closed

corridor of VR, respectively. Red triangles denote peaks of $Ca2^+$ signal. **b,** Representative $Ca2^+$ traces that peaked 2 to 6 sec after mice entered the center area (Post-center responsive cells). Red triangles indicate significant Ca^{2+} peaks. **c,** Number of categorized cell type that were sorted by the time mice spent crossing the center. Pre-center: Pre-center responsive cells; Post-center: Post-center responsive cells; Non-responsive: cells without response.

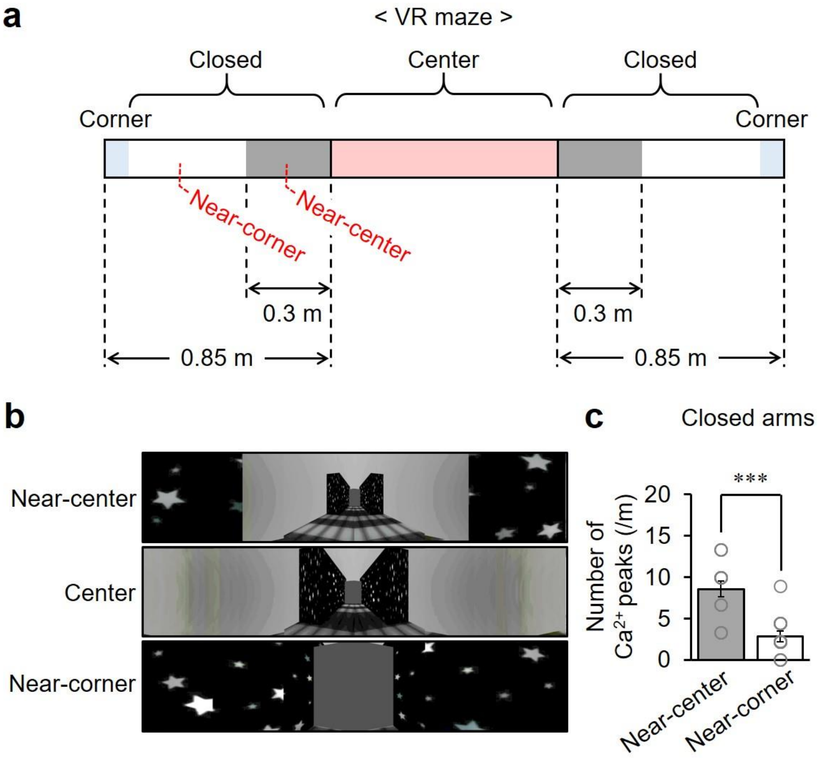

Supplementary figure 3. Astrocyte Ca^{2+} response in near-center and near-corner of VR maze. **a,** Illustration of VR condition in which closed area was divided by near-corner and near-center zones. **b,** Captured images of VR screen viewed by mouse at near-center, center, and near-corner zones. **c,** Number of calcium peaks per meter between near-center (n = 14 cells) and near-corner (n = 14 cells) VR zones. Two-tailed Mann-Whitney *U*-test ($p = 0.000$). Bar graphs are represented as mean ± SEM. ***, $p < 0.001$. See Supplementary Data for detailed statistics. Source data are provided as a Source Data file.

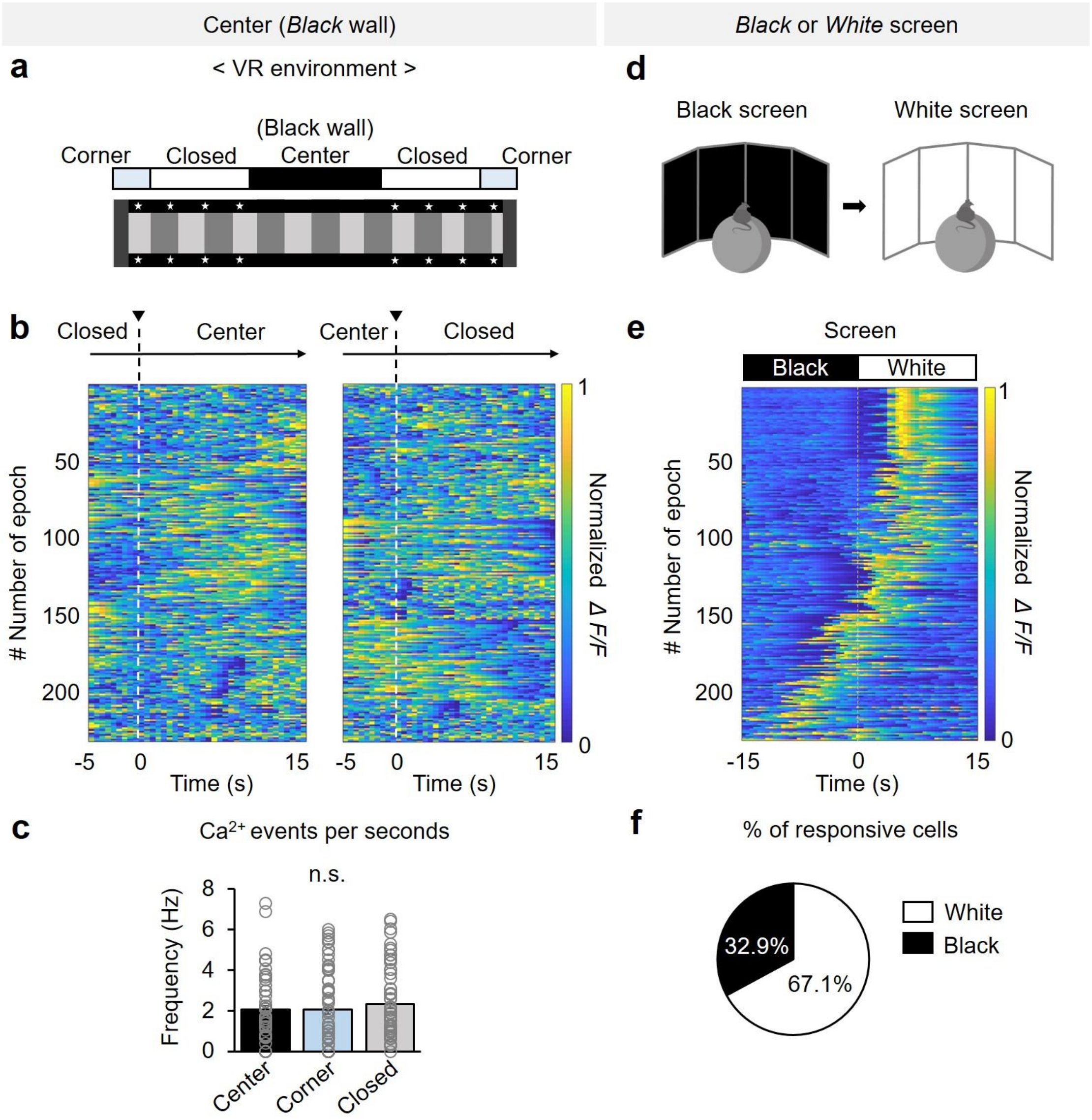

Supplementary figure 4. Astrocytes are activated upon exposure to visual-based anxiogenic environment in VR. a, Illustration of a VR condition in which the center area was changed to black-walled corridor. **b,** Heat map trace of normalized astrocytic GCaMP6s signals when mice entered the center from closed corridor (left) and when they went out to closed corridor from center (right; n = 222). Black arrow: Direction of movement. **c,** Number of Ca^{2+} peaks per second when mice were in black-walled center, corner or closed VR area. Number of calcium peaks when mice were in center (n = 64 cells), corner (n = 64 cells), and closed (n = 64 cells) areas. One-way

ANOVA, $p = 0.575$. **d,** Illustration of the VR conditions with whole black or white screen surrounding as seen by the mouse. **e,** Heat map trace of normalized astrocytic GCaMP6s signals when mice were exposed to black or white screen surrounding (n = 228 epochs). **f,** Percentage of the white-screen- and black-screen-responsive cells. The bar graphs depict the mean ± SEM. See Supplementary Table 1 for additional statistics. Bar graphs are represented as mean ± SEM. n.s., not significant. See Supplementary Data for detailed statistics. Source data are provided as a Source Data file.

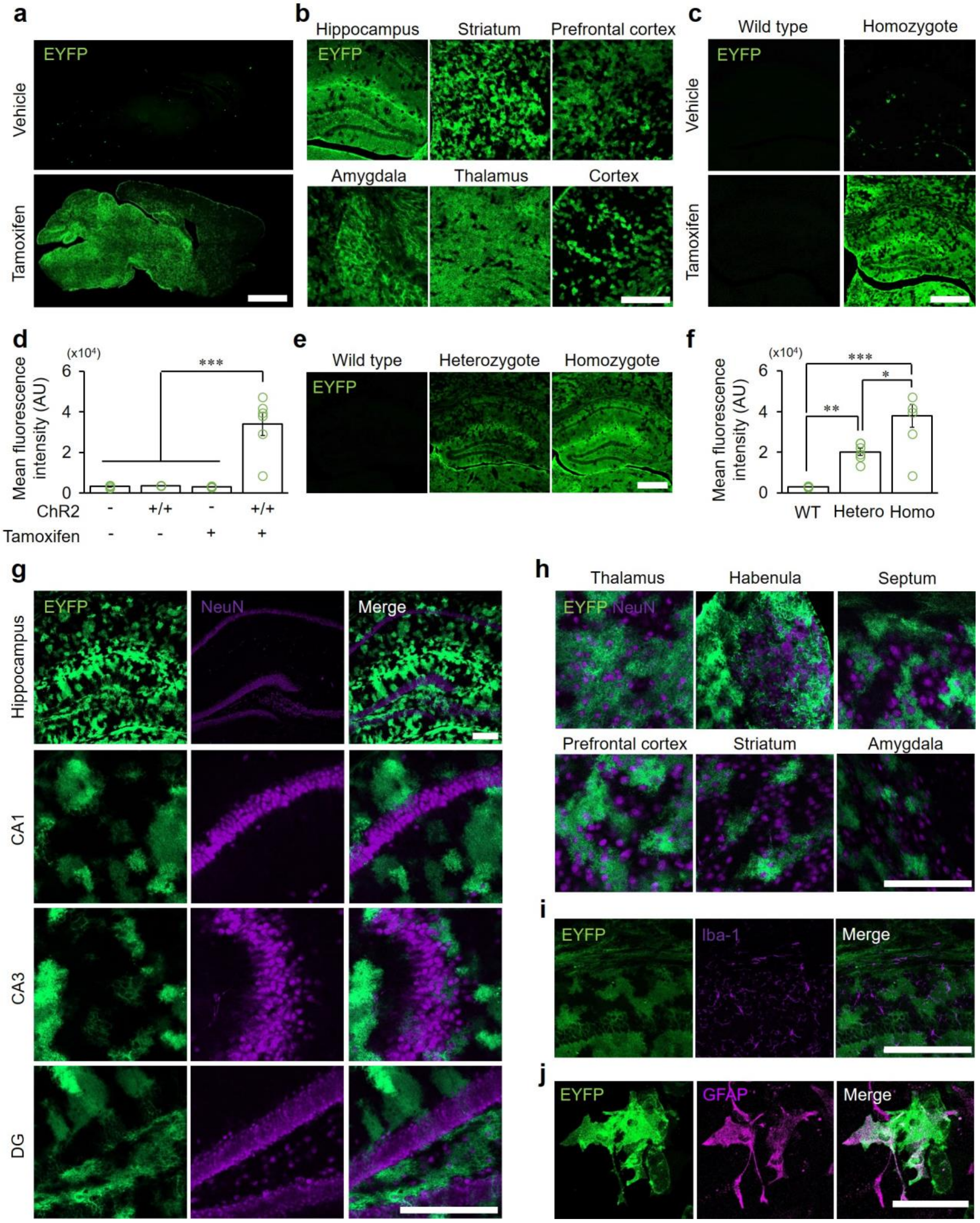

Supplementary figure 5. Astrocyte-specific ChR2 expression in hGFAP-ChR2 mice. a, Sagittal section of whole brain showing tamoxifen-induced EYFP (ChR2) expression. Scale bar, 2

mm. **b,** EYFP (green) expression in hippocampus, striatum, prefrontal cortex, amygdala, thalamus, and cortex of hGFAP-ChR2 mice. Scale bar, 500 μm. **c,** EYFP expression in hippocampus of wild-type and homozygote hGFAP-ChR2 mice after vehicle or tamoxifen injection. Scale bar, 500 μm. Representative data from three independent experiments (a-c). **d,** Mean fluorescence intensity of EYFP signal measured in hippocampal sections of vehicle-injected wild-type mice (n = 4), vehicle-injected homozygote hGFAP-ChR2 mice (n = 3), tamoxifen-injected wild-type mice (n = 5), and tamoxifen-injected homozygote hGFAP-ChR2 mice (n = 6). One-way ANOVA (p = 0.000) followed by post hoc analysis LSD (p = 0.000 (vehicle-injected wild-type vs tamoxifen-injected homozygote hGFAP-ChR2), p = 0.000 (vehicle-injected homozygote hGFAP-ChR2 vs tamoxifen-injected hGFAP-ChR2), p = 0.000 (tamoxifen-injected wild-type vs tamoxifen-injected hGFAP-ChR2)). **e,** EYFP expression in hippocampus of wild-type, heterozygote, and homozygote hGFAP-ChR2 mice. Scale bar, 500 μm. Representative data from three independent experiments. **f,** Mean EYFP intensity in wild-type (n = 5 mice), heterozygote (n = 6 mice), and homozygote hGFAP-ChR2 (n = 6 mice). One-way ANOVA (p = 0.000) followed by LSD (p = 0.006 (wild-type vs heterozygote), p = 0.000 (wild-type vs homozygote), p = 0.016 (heterozygote vs homozygote)). **g,** Representative images of ChR2-EYFP (green) with NeuN (purple) in hippocampal subregions. Scale bar, 200 μm. **h,** Various brain sub-regions. Green, $EYFP^+$ (ChR2); $Purple^+$, NeuN. Scale bar, 200 μm. **i,** Representative images of ChR2-EYFP (green) expression with Iba-1 (purple) in CA1 hippocampus. Scale bar, 200 μm **j.** EYFP expression in primary cultured hGFAP-ChR2 astrocytes after tamoxifen treatment. Green, $EYFP^+$ (ChR2); Purple, $GFAP^+$. Scale bar, 100 μm. The bar graphs depict mean ± SEM. Representative data from three independent experiments (g-j). *, $p < 0.05$; **, $p < 0.01$; ***, $p < 0.001$. See Supplementary Data for detailed statistics. Source data are provided as a Source Data file.

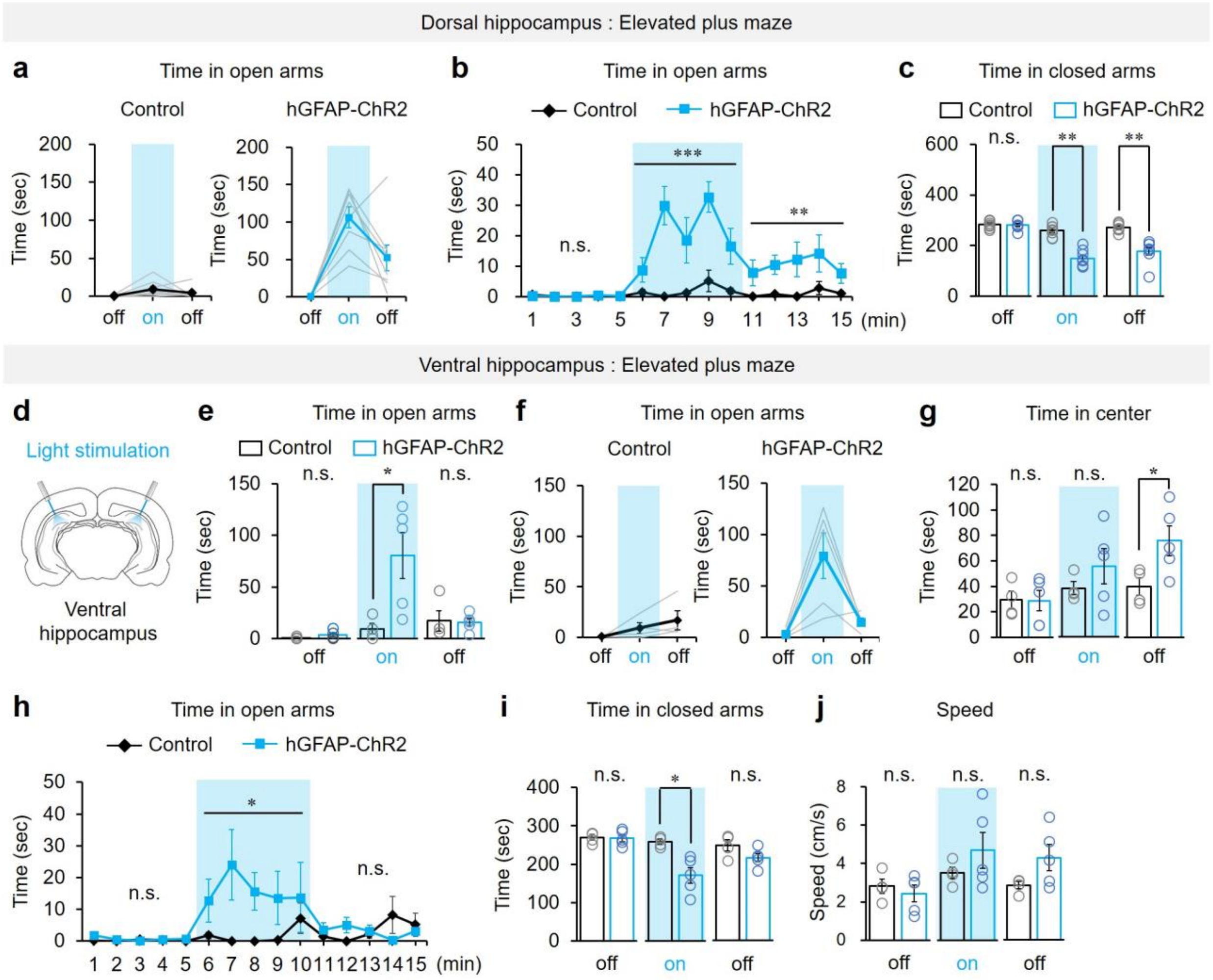

Supplementary figure 6. Optogenetic stimulation of astrocytes in dorsal and ventral hippocampi during EPM test. (a-c) EPM test with optogenetic stimulation of dorsal hippocampal astrocytes between control and hGFAP-ChR2 (n = 8 mice in each group). Time in open/closed arm is analyzed from this experiment. **a,** Time spent in the open arm between control and hGFAP-ChR2. **b,** Time spent in the open arm measured every min during a 15-min session. Two-way repeated measure ANOVA (p = 0.000 (light-on), p = 0.016 (last light-off)). **c,** Time spent in closed arm. Two-way repeated measure ANOVA (p = 0.000) followed by two-tailed Mann-Whitney U-test (p = 0.001 (light-on), p = 0.001 (last light-off)). **(d-j)** EPM test with optogenetic stimulation of ventral hippocampal astrocytes between control (n = 4 mice) and hGFAP-ChR2 (n = 5 mice).

Time in open/closed arm, time in center, and speed are analyzed from this experiment. **d,** Schematic illustration of optic fiber implantation and light stimulation in the ventral hippocampus. **e-f,** Time spent in the open arm during light stimulation of ventral hippocampus of control and hGFAP-ChR2. Two-way repeated measure ANOVA (p = 0.048) followed by two-tailed Mann-Whitney U-test (p = 0.027 (light-on)). Gray lines indicate individual mouse. **g,** Time spent in center during light stimulation of ventral hippocampus of control and hGFAP-ChR2. Two-way repeated measure ANOVA (p = 0.001) followed by two-tailed Mann-Whitney U-test (p = 0.050 (last light-off)). **h,** Time spent in the open arm during light stimulation of ventral hippocampus of control and hGFAP-ChR2 that measured every min during a 15 min session. Two-way repeated measure ANOVA (p = 0.029 (light-on)). **i,** Time spent in closed arm during light stimulation of ventral hippocampus of control and hGFAP-ChR2. Two-way repeated measure ANOVA (p = 0.001) followed by two-tailed Mann-Whitney U-test (p = 0.014 (light-on)). **j,** Travel speed during light stimulations of ventral hippocampus of control and hGFAP-ChR2. Two-way repeated measure ANOVA. The bar and line graphs depict data as mean ± SEM. *, $p < 0.05$; **, $p < 0.01$; ***, $p < 0.001$; n.s., not significant. See Supplementary Data for detailed statistics. Source data are provided as a Source Data file.

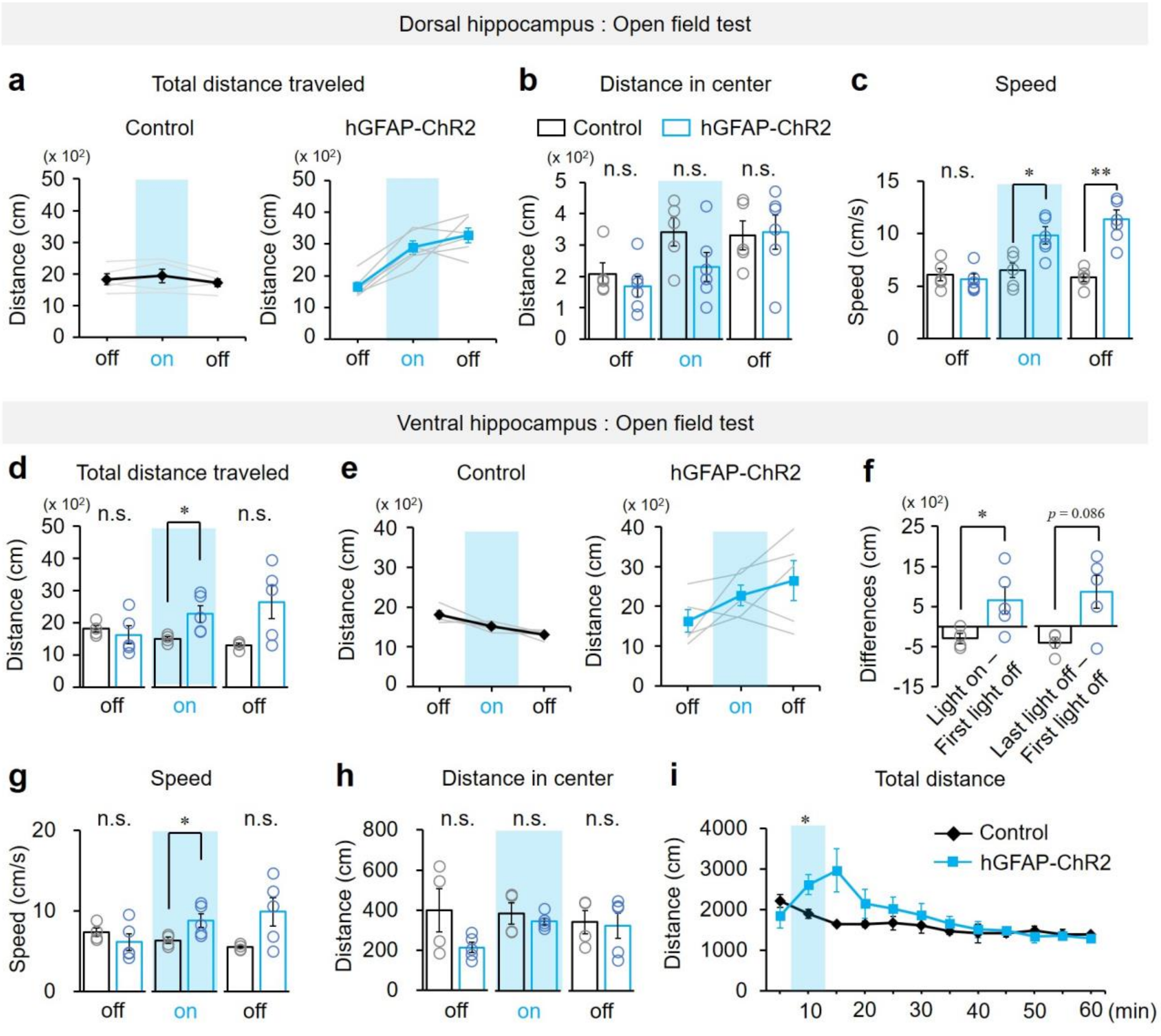

Supplementary figure 7. Optogenetic stimulation of astrocytes in dorsal and ventral hippocampi during OFT. (a-c) OFT test with optogenetic stimulation of dorsal hippocampal astrocytes between control (n = 5 mice) and hGFAP-ChR2 (n = 6 mice). Total distance traveled, distance in center, and speed are analyzed from this experiment. **a,** Total distance travelled between control and hGFAP-ChR2. **b,** Distance traveled in center between control and hGFAP-ChR2. Two-way repeated measure ANOVA. **c,** Travel speed between control and hGFAP-ChR2. Two-way repeated measure ANOVA (p = 0.007) followed by two-tailed Mann-Whitney U-test (p = 0.018 (light-on), p = 0.006 (last light-off)). **(d-i)** OFT test with optogenetic stimulation of ventral

hippocampal astrocytes between control (n = 4 mice) and hGFAP-ChR2 (n = 5 mice). Total distance traveled, distance in center, speed, and differences are analyzed from this experiment. **d,** Distance traveled in the open field test between control and hGFAP-ChR2. Two-way repeated measure ANOVA followed by two-tailed Mann-Whitney *U*-test ($p = 0.027$ (light-on)). **e,** Total distance traveled between control and hGFAP-ChR2. **f,** Differences in distance traveled between ventral hippocampus stimulation of control and hGFAP-ChR2 during light-on epoch and the first light-off epoch (light-on – first light-off) and during the last light-off epoch and the first light-off epoch (last light-off – first light-off). Two-tailed Mann-Whitney *U*-test ($p = 0.050$ (light-on – first light-off)). **g,** Travel speed between control and hGFAP-ChR2. Two-way repeated measure ANOVA followed by two-tailed Mann-Whitney *U*-test ($p = 0.027$ (light-on)). **h,** Distance traveled in the center between control and hGFAP-ChR2. Two-way repeated measure ANOVA. **i,** Total distance traveled between control and hGFAP-ChR2 measured every 5 min during the 1-h OFT session. Two-tailed Mann-Whitney *U*-test ($p = 0.027$ (5-10 min)). The bar and line graphs depict the mean ± SEM. *, $p < 0.05$; **, $p < 0.01$; n.s., not significant. See Supplementary Data for detailed statistics. Source data are provided as a Source Data file.

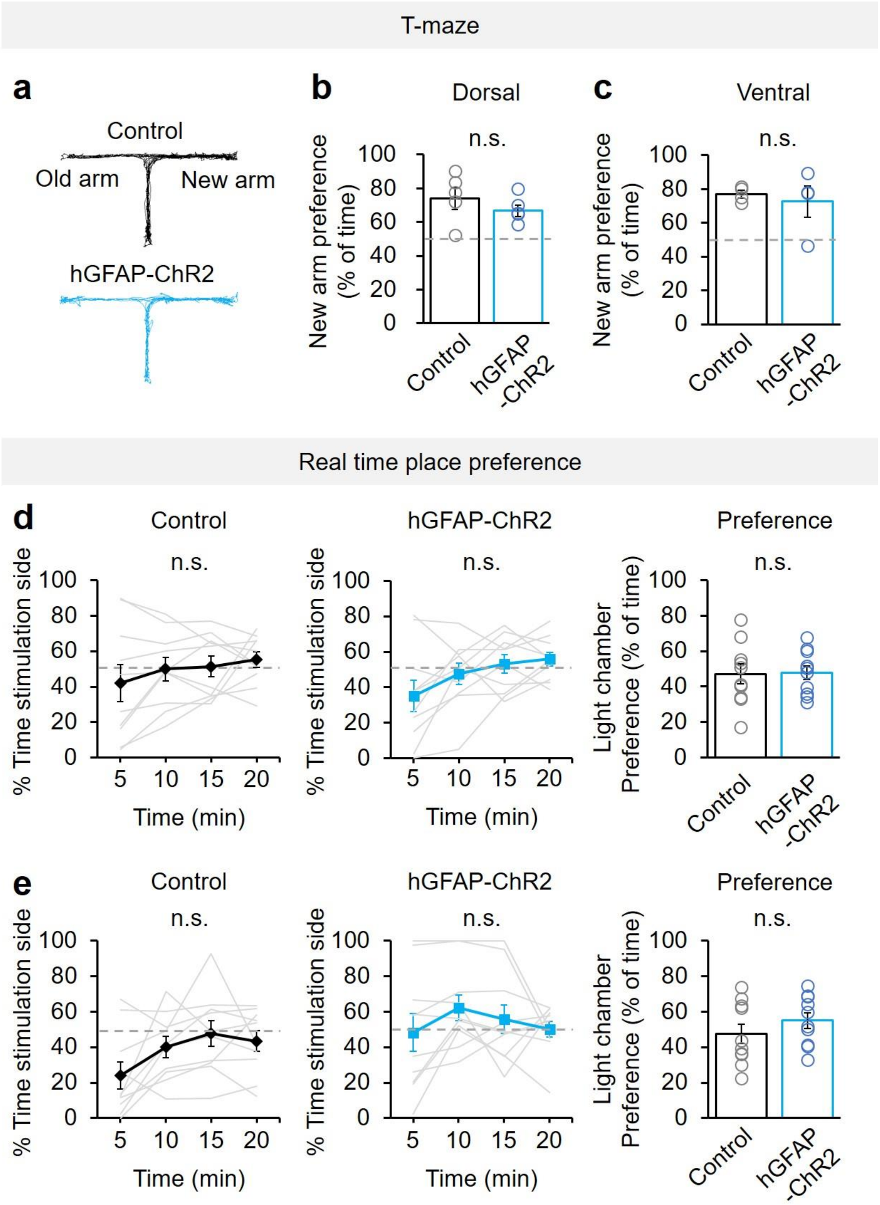

Supplementary figure 8. Optogenetic hippocampal astrocyte stimulation during T-maze and real time place preference (RTPP) tests. Mice were exposed to start arm and old arm for 20 min with light stimulation during the first 5 min either in the dorsal (b) or ventral (c) hippocampus, and then re-exposed to the entire T-maze after 5 min. **a,** Representative trace of mouse movement in T-maze (control, upper; hGFAP-ChR2, bottom). **b,** New arm preference in T-maze during light

stimulation of dorsal hippocampus of control and hGFAP-ChR2 (n = 5 mice in each group). Two-tailed Mann-Whitney *U*-test ($p = 0.251$). **c,** New arm preference in T-maze during light stimulation of ventral hippocampus of control and hGFAP-ChR2 (n = 6 mice in each group). Two-tailed Mann-Whitney *U*-test ($p = 1.000$). **d,** Percentage of time spent by control (left) and hGFAP-ChdR2 (middle) (n = 10 mice in each group) in the dorsal hippocampus stimulation-paired chamber measured every 5 min during the 20-min session. Two-way repeated measure ANOVA ($p = 0.824$). Light chamber preference by percentage of time (right) for 15 min without stimulation. Two-tailed Mann-Whitney *U*-test ($p = 1.000$). **e,** Percentage of time spent by control (left) and hGFAP-ChdR2 (middle) (n = 10 mice in each group) in the ventral hippocampus stimulation-paired chamber measured every 5 min during the 20-min session. Two-way repeated measure ANOVA ($p = 0.080$). Light chamber preference by percentage of time (right) for15 min without stimulation. Two-tailed Mann-Whitney *U*-test ($p = 0.226$). The bar graphs depict the mean ± SEM. n.s., not significant. See Supplementary Data for detailed statistics. Source data are provided as a Source Data file.

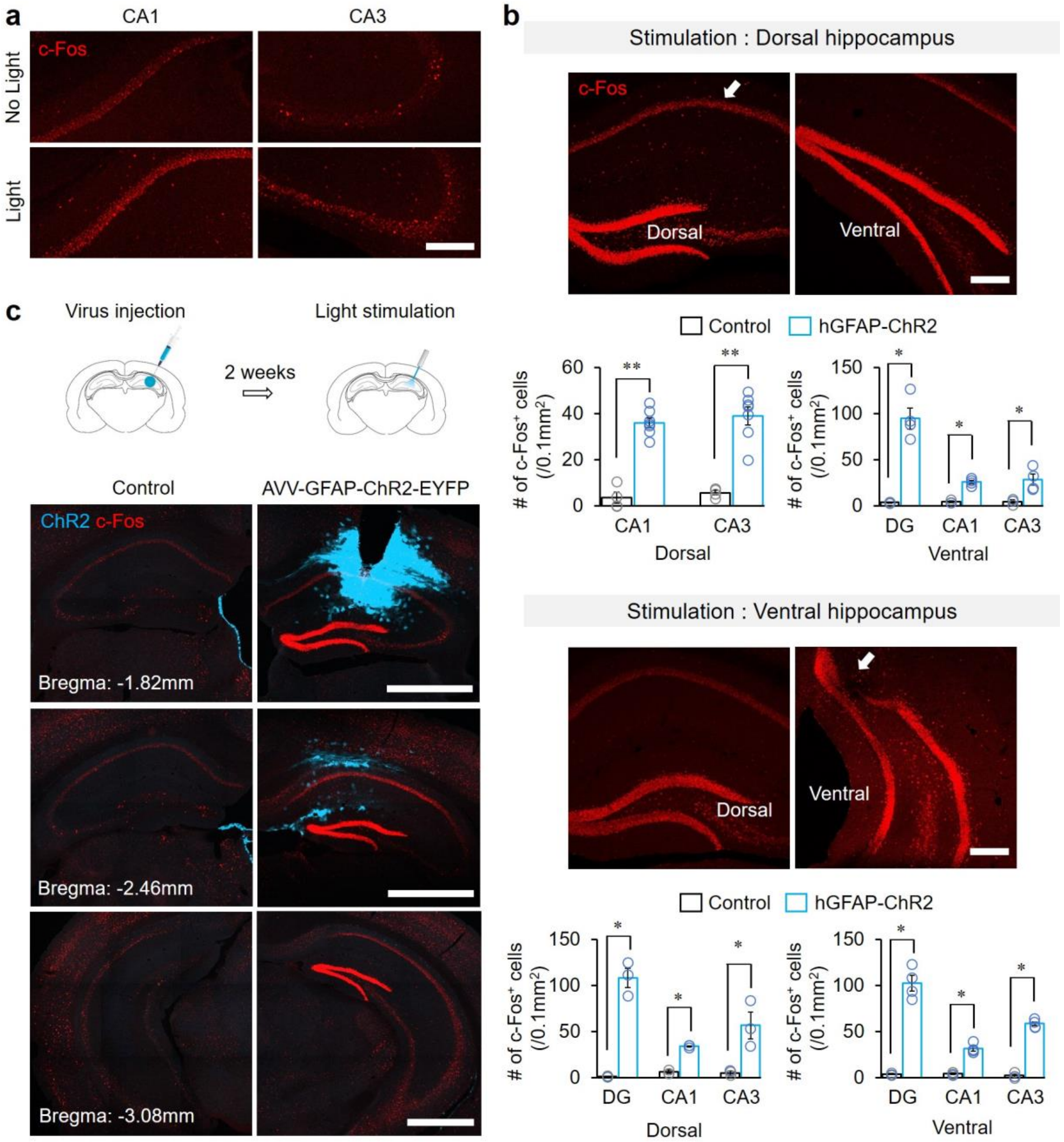

Supplementary figure 9. Expression of c-Fos in dorsal and ventral hippocampus after optogenetic stimulation of dorsal and ventral hippocampal astrocytes. a, Light-induced astrocyte activation increased c-Fos (red) expression in the CA1 and CA3 areas of the hippocampus. Scale bar, 200 μm. Representative data from three independent experiments. **b,** Representative images of c-Fos expression and implantation of optic fiber in dorsal (upper) and

ventral hippocampus (lower). White arrow: Track of implantation. Scale bar, 200 μm. Quantification of the number of c-Fos positive cells in dorsal (left) and ventral (right) hippocampi induced by dorsal hippocampus astrocyte stimulation (upper) and vice versa with ventral region (lower) (dorsal CA1 and dorsal CA3 in dorsal hippocampus stimulation: control (n = 4 mice), hGFAP-ChR2 (n = 7 mice); ventral DG, ventral CA1, ventral CA3: control (n = 3 mice), hGFAP-ChR2 (n = 3 mice)) (dorsal DG, dorsal CA1, and dorsal CA3 in ventral hippocampus stimulation: control (n = 3 mice), hGFAP-ChR2 (n = 4 mice); ventral DG, ventral CA1, ventral CA3: control (n = 3 mice), hGFAP-ChR2 (n = 4 mice)). Two-tailed Mann-Whitney *U*-test (p = 0.008 (dorsal implant-dorsal CA1), p = 0.008 (CA3), p = 0.050 (dorsal implant-ventral DG), p = 0.046 (CA1), p = 0.050 (CA3), p = 0.032 (ventral implant-dorsal DG), p = 0.032 (CA1), p = 0.034 (CA3), p = 0.034 (ventral implant-ventral DG), p = 0.034 (CA1), p = 0.034 (CA3)). **c,** Schematic diagram of AAV-GFAP-ChR2-EYFP virus injection and light stimulation 2 weeks after surgery (upper) and the representative confocal images (lower) of light induced astrocyte c-Fos (red) expression in DG hippocampal regions. Blue, ChR2-EYFP. Scale bar, 1 mm. Representative data from three independent experiments. Bar graphs depict data as mean ± SEM. *, $p < 0.05$; **, $p < 0.01$. See Supplementary Data for detailed statistics. Source data are provided as a Source Data file.

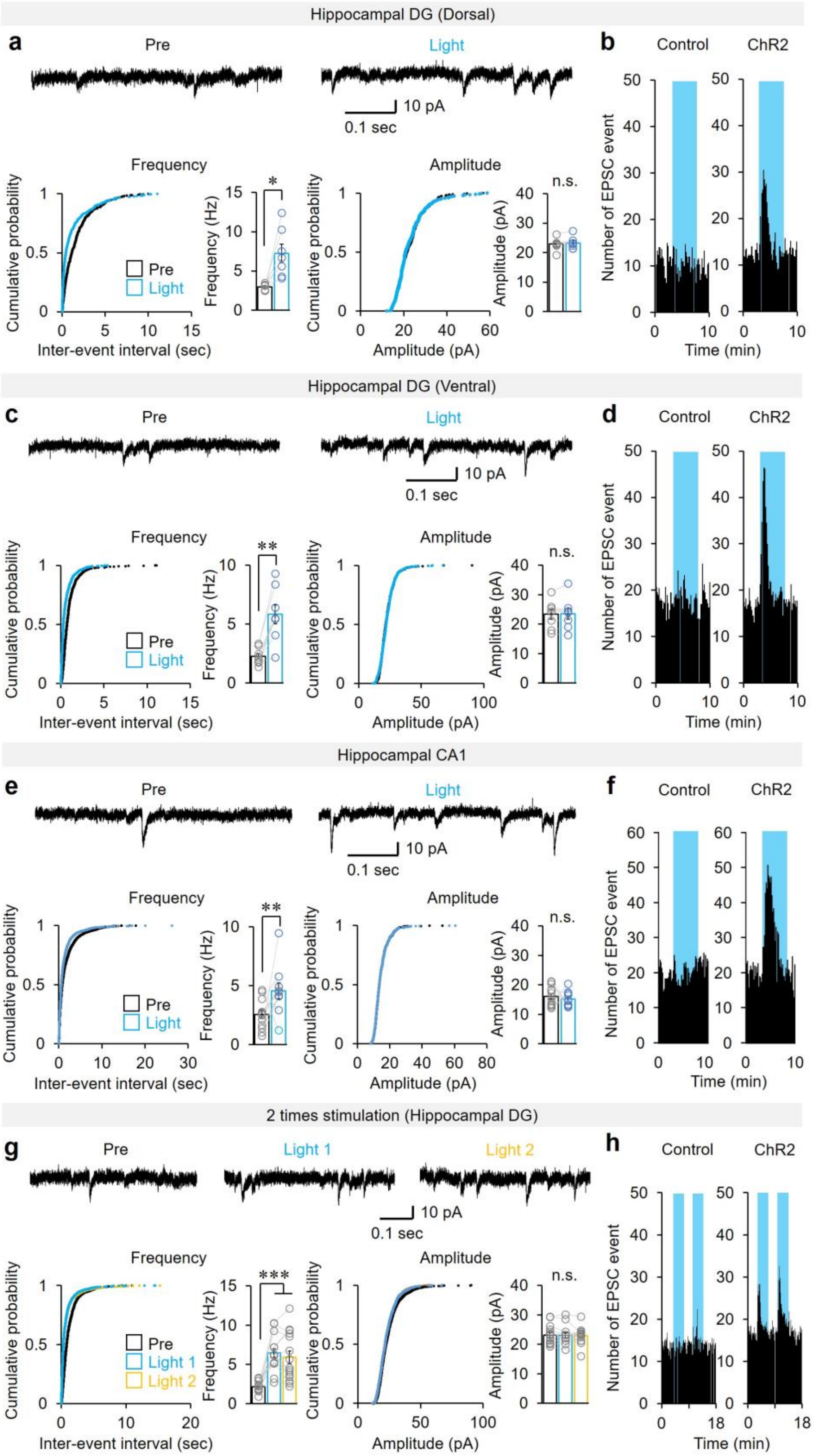

Hippocampal DG (Dorsal)
a
Pre
Light
10 pA
0.1 sec
Frequency
Cumulative probability
Pre
Light
Inter-event interval (sec)
Frequency (Hz)
*
Amplitude
Amplitude (pA)
n.s.
b
Control
ChR2
Number of EPSC event
Time (min)
Hippocampal DG (Ventral)
c
d
**
Hippocampal CA1
e
f
2 times stimulation (Hippocampal DG)
g
Light 1
Light 2
***
h

Supplementary figure 10. Excitatory synaptic transmissions upon optogenetic hippocampal astrocytes stimulation. a, Upper, Representative sEPSC traces measured from dorsal hippocampus DG. Bottom, sEPSC frequency and amplitude of hGFAP-ChR2 (n = 7 cells). Paired t-test for sEPSC frequency (p = 0.010) and amplitude (p = 0.627). Two-sample Kolmogorov-Smirnov test for cumulative probability of frequency (p = 0.000) and amplitude (p = 0.761). **b,** Number of sEPSC events. **c,** Upper, Representative sEPSC traces measured from ventral hippocampus DG. Bottom, sEPSC frequency and amplitude of hGFAP-ChR2 (n = 8 cells). Paired t-test for sEPSC frequency (p = 0.001) and amplitude (p = 0.779). Two-sample Kolmogorov-Smirnov test for cumulative probability of frequency (p = 0.000) and amplitude (p = 0.554). **d,** Number of sEPSC events. **e,** Upper, Representative sEPSC traces measured from hippocampus CA1. Bottom, sEPSC frequency and amplitude of hGFAP-ChR2 (n = 10 cells). Paired t-test for sEPSC frequency (p = 0.005) and amplitude (p = 0.562). Two-sample Kolmogorov-Smirnov test for cumulative probability of frequency (p = 0.046) and amplitude (p = 1.000). **f,** Number of sEPSC events. **g,** Upper, Representative sEPSC traces measured from hippocampus DG. Bottom, sEPSC frequency and amplitude of hGFAP-ChR2 during pre, light 1, and light 2 stimulations (n = 15 cells). One-way ANOVA for frequency (p = 0.000) followed by Bonferroni post hoc analysis (p = 0.000 (pre vs light 1), p = 0.000 (pre vs light 2), p = 1.000 (light 1 vs light 2)). Two-sample Kolmogorov-Smirnov test for cumulative probability of frequency (p = 0.000 (pre vs light 1), p = 0.000 (pre vs light 2), p = 0.000 (light 1 vs light 2)). One-way ANOVA for amplitude (p = 0.984). Two-sample Kolmogorov-Smirnov test for cumulative probability (p = 0.124 (pre vs light 1), p = 0.000 (pre vs light 2), p = 0.000 (light 1 vs light 2)). **h,** Number of sEPSC events. Scale bar, 0.1 sec and 10 pA. Bar graphs depict data as mean ± SEM. **, p < 0.01; ***, p < 0.001; n.s., not significant. See Supplementary Data for detailed statistics. Source data are provided as a Source

Data file.

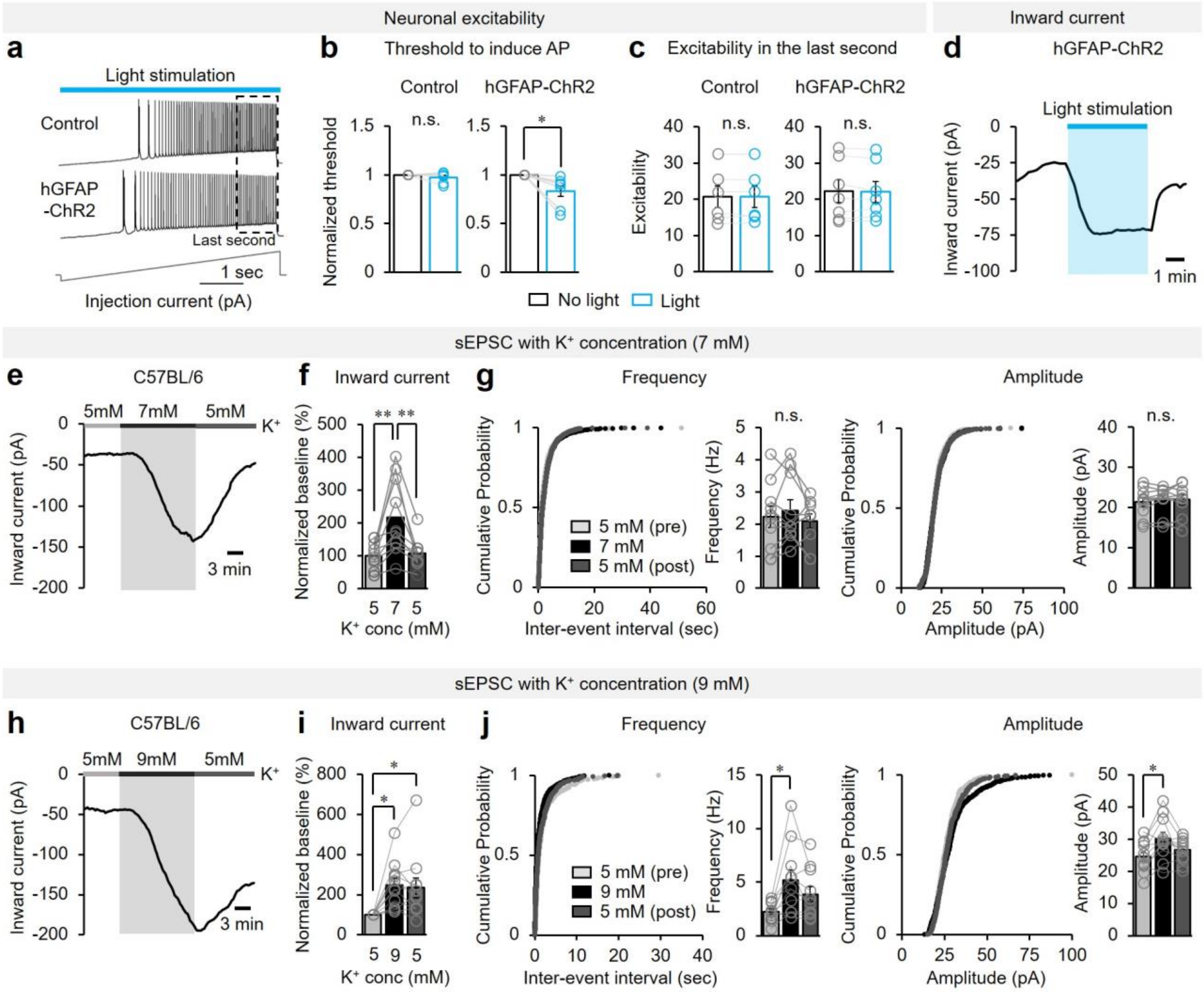

Supplementary figure 11. Neuronal excitability upon optogenetic astrocytes stimulation and inward currents upon extracellular K^+ increase. a, Sample traces showing AP firing during light stimulation. Scale bar, 1 sec. Dashed lines depict the last second recorded traces used in (c). **b,** Normalized threshold current to induce AP between control (n = 6 cells) and hGFAP-ChR2 (n = 7 cells). Paired t-test (p = 0.030 (hGFAP-ChR2: no light vs light)). **c,** Excitability in the last second between control (n = 6 cells) and hGFAP-ChR2 (n = 7 cells). Paired t-test. **d,** Mean baseline inward current of hippocampal granule cell by optogenetic astrocyte stimulation. **e,** Mean baseline inward current of hippocampal granule cell by changing K^+ concentrations in ACSF buffer in C57BL/6 mice. **f,** Quantification of baseline inward currents (n = 10 cells in each group). One-way

ANOVA (p = 0.003) followed by Bonferroni post hoc analysis (p = 0.005 (first 5 mM vs 7 mM), p = 0.009 (7 mM vs last 5 mM)). Amplitude: One-way ANOVA. **g,** sEPSC frequency and amplitude (n = 10 cells in each group). Frequency: One-way ANOVA followed by Bonferroni post hoc analysis. Cumulative probabilities were compared using Two-sample Kolmogorov-Smirnov test. Amplitude: One-way ANOVA followed by Bonferroni post hoc analysis. Cumulative probabilities were compared using Two-sample Kolmogorov-Smirnov test. **h,** Mean baseline inward current of C57BL/6 hippocampal granule cells with K^+ concentrations. **i,** Quantification of baseline inward currents (n = 11 cells in each group). One-way ANOVA (p = 0.009) followed by Bonferroni post hoc analysis (p = 0.016 (first 5 mM, vs 9 mM), p = 0.010 (first 5 mM vs last 5 mM)). **j,** sEPSC frequency and amplitude (n = 11 cells in each group). Frequecy: One-way ANOVA (p = 0.020) followed by Bonferroni post hoc analysis (p = 0.016 (first 5 mM vs 9 mM)). Cumulative probabilities were compared using Two-sample Kolmogorov-Smirnov test. Amplitude: One-way ANOVA (p = 0.055) followed by Bonferroni post hoc analysis (p = 0.050 (first 5 mM vs 9 mM)). Cumulative probabilities were compared using Two-sample Kolmogorov-Smirnov test. Bar graphs depict data as mean ± SEM. *, $p < 0.05$; **, $p < 0.005$; n.s., not significant. See Supplementary Data for detailed statistics. Source data are provided as a Source Data file.

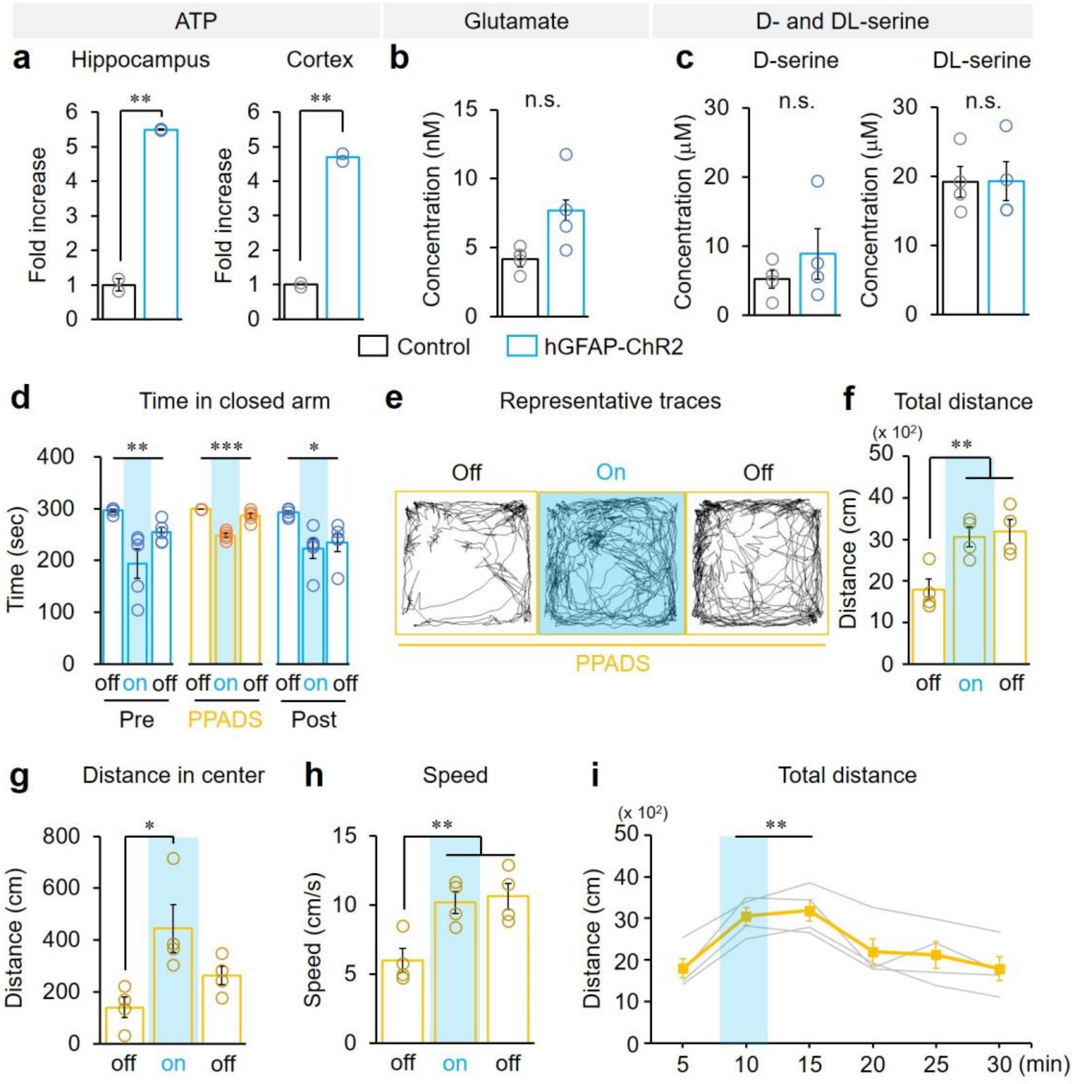

Supplementary figure 12. Gliotransmitter assays and OFT with optogenetic astrocytes stimulation while PPADS treatment. a, Fold increase of ATP release from hippocampal (left) and cortical primary astrocytes (right) between control and hGFAP-ChR2 (n = 2 mice in each group). Independent two sample t-test (p = 0.020 (hippocampus), p = 0.001 (cortex)). **b,** Glutamate release from control and hGFAP-ChR2 (n = 4 mice in each group). Independent two sample t-test (p = 0.063). **c,** D-serine (left) and DL-serine (right) release from control and hGFAP-ChR2 (n = 3 mice in each group). Independent two sample t-test (p = 0.444 (D-serine), p = 0.922 (DL-serine)). **d,** Time spent in EPM closed arm during Pre/PPADS/Post (n = 5 mice in each group) treatments

during light stimulation epochs. One-way ANOVA for Pre treatment (p = 0.004) followed by post hoc analysis LSD. **(e-i)** OFT with optogenetic astrocytes stimulation while PPADS treatment in hGFAP-ChR2 mice (n = 4 mice). Total distance, distance in center, and speed are analyzed from this experiment. **e,** Representative traces of hGFAP-ChR2 mouse movement in the OFT with PPADS treatment. **f,** Total distance traveled in OFT during PPADS treatment and light stimulation. Paired t-test (p = 0.007 (first light-off vs light-on), p = 0.001 (first light-off vs last light-off)). **g,** Distance traveled in the center of OFT during PPADS treatment and light stimulation. Paired t-test (p = 0.047 (first light-off vs light-on), p = 0.053 (first light-off vs last light-off)). **h,** Travel speed in OFT during PPADS treatment and light stimulations. Paired t-test (p = 0.007 (first light-off vs light-on), p = 0.001 (first light-off vs last light-off)). **i,** Distance traveled measured every 5 min during the 30-min OFT. Paired t-test (p = 0.007 (0-5 min vs 5-10 min), p = 0.001 (0-5min vs 10-15 min)). Bar graphs depict data as mean ± SEM. *, $p < 0.05$; **, $p < 0.005$; ***, $p < 0.001$; n.s., not significant. See Supplementary Data for detailed statistics. Source data are provided as a Source Data file.

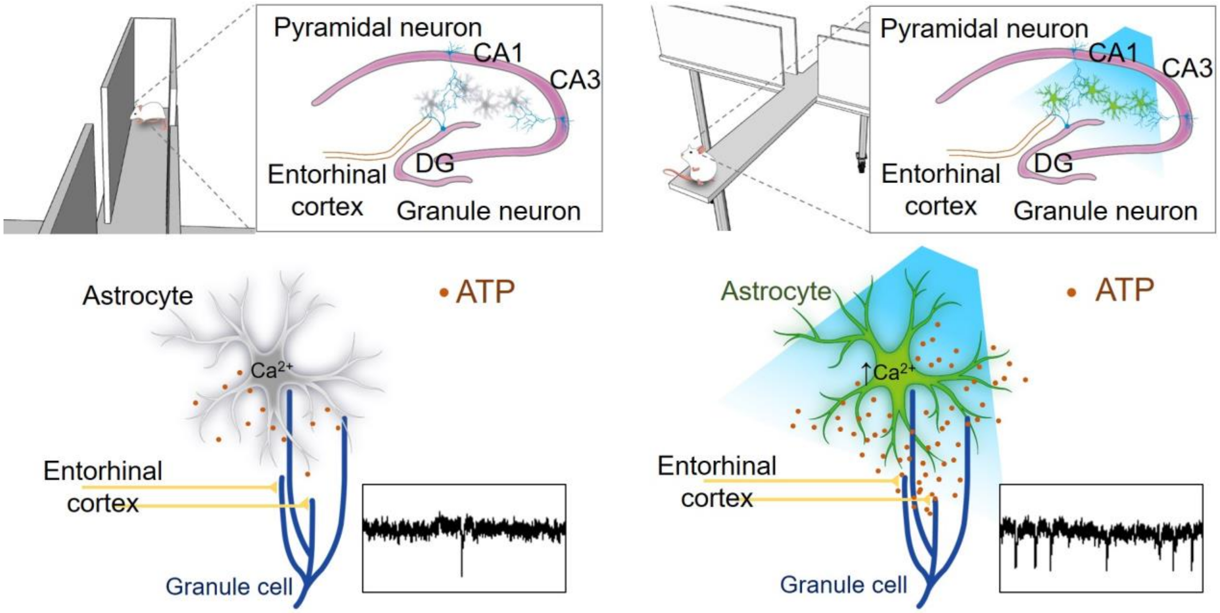

Supplementary figure 13. Schematic illustration showing that optogenetically activated hippocampal astrocytes regulate anxiety-like behavior by releasing ATP
