# Supplementary material for: Hippocampal astrocytes modulate anxiety-like behavior
Source: Nat Commun. 2022 Nov 7;13:6536. doi: 10.1038/s41467-022-34201-z (PMC9640657; doi:10.1038/s41467-022-34201-z)
Supplement: Supplementary file 3 — Description of Additional Supplementary Files [file 41467_2022_34201_MOESM3_ESM.docx]

**Description of Additional Supplementary Files**

**File Name**: Supplementary Data

**Description**: Statistical table providing detailed statistical values and tests used.
