# Supplementary material for: Hippocampal astrocytes modulate anxiety-like behavior
Source: Nat Commun. 2022 Nov 7;13:6536. doi: 10.1038/s41467-022-34201-z (PMC9640657; doi:10.1038/s41467-022-34201-z)
Supplement: Supplementary file 5 — Reporting Summary [file 41467_2022_34201_MOESM5_ESM.pdf]

nature portfolio

Corresponding author(s): Sung Joong Lee

Last updated by author(s): Sept 27, 2022

Reporting Summary

Nature Portfolio wishes to improve the reproducibility of the work that we publish. This form provides structure for consistency and transparency in reporting. For further information on Nature Portfolio policies, see our Editorial Policies and the Editorial Policy Checklist.

Statistics

For all statistical analyses, confirm that the following items are present in the figure legend, table legend, main text, or Methods section.

n/a	Confirmed	
☐	☒	The exact sample size (n) for each experimental group/condition, given as a discrete number and unit of measurement
☐	☒	A statement on whether measurements were taken from distinct samples or whether the same sample was measured repeatedly
☐	☒	The statistical test(s) used AND whether they are one- or two-sided<br>*Only common tests should be described solely by name; describe more complex techniques in the Methods section.*
☒	☐	A description of all covariates tested
☐	☒	A description of any assumptions or corrections, such as tests of normality and adjustment for multiple comparisons
☐	☒	A full description of the statistical parameters including central tendency (e.g. means) or other basic estimates (e.g. regression coefficient) AND variation (e.g. standard deviation) or associated estimates of uncertainty (e.g. confidence intervals)
☐	☒	For null hypothesis testing, the test statistic (e.g. F, t, r) with confidence intervals, effect sizes, degrees of freedom and P value noted<br>*Give P values as exact values whenever suitable.*
☒	☐	For Bayesian analysis, information on the choice of priors and Markov chain Monte Carlo settings
☒	☐	For hierarchical and complex designs, identification of the appropriate level for tests and full reporting of outcomes
☒	☐	Estimates of effect sizes (e.g. Cohen's d, Pearson's r), indicating how they were calculated

Our web collection on statistics for biologists contains articles on many of the points above.

Software and code

Policy information about availability of computer code

Data collection	NoRMCorre Software and custom code (run on Matlab v2020b) , CalmAn Software (v5), Zeiss Zen (v2.0), SMART 3.0 Panlab Harvard Apparatus, EZcalcium Software (run on Matlab v2020b)
Data analysis	MetaFluor Software (v7), MathWorks MATLAB(v2020b), Zeiss ZEN (v2.0), SPSS(v25)

For manuscripts utilizing custom algorithms or software that are central to the research but not yet described in published literature, software must be made available to editors and reviewers. We strongly encourage code deposition in a community repository (e.g. GitHub). See the Nature Portfolio guidelines for submitting code & software for further information.

Data

Policy information about availability of data

All manuscripts must include a data availability statement. This statement should provide the following information, where applicable:

- Accession codes, unique identifiers, or web links for publicly available datasets
- A description of any restrictions on data availability
- For clinical datasets or third party data, please ensure that the statement adheres to our policy

All data generated in this study are provided in the Supplementary Information and Source Data file. The raw data images that support the findings of the current study are available from the corresponding author upon reasonable request. Source data are provided with this paper.

Field-specific reporting

Please select the one below that is the best fit for your research. If you are not sure, read the appropriate sections before making your selection.

☒ Life sciences ☐ Behavioural & social sciences ☐ Ecological, evolutionary & environmental sciences

For a reference copy of the document with all sections, see nature.com/documents/nr-reporting-summary-flat.pdf

Life sciences study design

All studies must disclose on these points even when the disclosure is negative.

Sample size	No statistical methods were used to predetermine sample size. Common mouse sample sizes (at least n = 5 for behavioral and molecular experiments, at least n = 3 for histology) were used. Sample sizes used were based on our previous publications (Oh et al., 2022, Sci Rep; Noh et al., 2019, Mol Psychiatry)
Data exclusions	Implant placements were verified by histology before the data were included in the analysis.
Replication	Behavioral experiments (calcium imaging, optogenetics, and molecular experiments were independently replicated twice with each set including sufficient number of animals similar to previous studies. Main effects and findings were successfully replicated.
Randomization	Mice were randomly assigned to experimental or control groups before surgery and experiments.
Blinding	Blinding was used to analyze and quantify histology data. All other analyses were conducted by automated software with experimenters not blinded to conditions.

Reporting for specific materials, systems and methods

We require information from authors about some types of materials, experimental systems and methods used in many studies. Here, indicate whether each material, system or method listed is relevant to your study. If you are not sure if a list item applies to your research, read the appropriate section before selecting a response.

Materials & experimental systems

n/a	Involved in the study	
☐	☒	Antibodies
☒	☐	Eukaryotic cell lines
☒	☐	Palaeontology and archaeology
☐	☒	Animals and other organisms
☒	☐	Human research participants
☒	☐	Clinical data
☒	☐	Dual use research of concern

Methods

n/a	Involved in the study	
☒	☐	ChIP-seq
☒	☐	Flow cytometry
☒	☐	MRI-based neuroimaging

Antibodies

Antibodies used	Mouse anti-NeuN, clone A60 (MAB377B, 1:1,000; Millipore, Billerica, MA, USA)<br>Mouse anti-GFAP, clone GA5 (MAB360, 1:1,000; Millipore)<br>Rabbit anti-S100b, clone EP1576Y (ab52642, 1:500; Abcam, Cambridge, MA, USA)<br>Rabbit anti-Iba1 (019-19741, 1:1,000; Wako, Richmond, VA, USA)<br>Rabbit anti-c-Fos (PC05-100UG, 1:1,000; Millipore)<br>Donkey, anti-Rabbit FITC (711-095-152), anti-Rabbit Cy3 (711-165-152), anti-Rabbit Cy5 (711-175-152), anti-Mouse FITC (715-195-151), anti-Mouse Cy3 (115-165-003), anti-Mouse Cy5 (715-175-150) secondary antibodies (All 1:200, All from Jackson ImmunoResearch Laboratory).
Validation	Mouse anti-NeuN, clone A60 (MAB377B, 1:1,000; Millipore): > 4,325 citations,<br>Validation from manufacturer's data sheet: validated for mouse/ rat IHC and ICC. 1:200<br>(https://www.sigmaaldrich.com/KR/en/search/mab377)<br>Mouse anti-GFAP, clone GA5 (MAB360, 1:1,000; Millipore): 65 citations,<br>Validation from manufacturer's data sheet: validated for chicken, pig, mouse, rat, rabbit, bovine, human for IHC and ICC<br>(https://www.sigmaaldrich.com/KR/en/product/mm/mab360)<br>Rabbit anti-S100b, clone EP1576Y (ab52642, 1:500; Abcam): 219 citations,<br>Validation from manufacturer's data sheet: validated for mouse, rat, human IHC and ICC/IF<br>(https://www.abcam.com/S100-beta-antibody-EP1576Y-Astrocyte-Marker-ab52642)<br>Rabbit anti-Iba1 (019-19741, 1:1,000; Wako): 3,287 citations<br>Validation from manufacturer's data sheet: validated for mouse, rat, human IHC and ICC<br>(https://labchem-wako.fujifilm.com/us/product/detail/W01W0101-1974)

Rabbit anti-c-Fos (PC05-100UG, 1:1,000; Millipore), 1 citation,
Validation from manufacturer's data sheet: validated for mouse, rat, human IHC and ICC
(https://www.merckmillipore.com/KR/en/product/Anti-c-Fos-Ab-2-4-17-Rabbit-pAb,EMD_BIO-PC05)

Animals and other organisms

Policy information about studies involving animals; ARRIVE guidelines recommended for reporting animal research

Laboratory animals	Male 8 to 16 week-old C57BL/6J mice (from DooYeol Biotech Co., Seoul, Korea) and male, 8 to 12 week-old, Floxed-GCaMP6s and Floxed-ChR2(H134R)-EYFP mice (Jackson Laboratory, Stock no. 028866 & 12569, Bar Harbor, ME, USA), hGFAP-CreERT2 mice (from the laboratory of Dr. Frank Kirchhoff, Max Plank Institute, Munich, Germany), Floxed-GCaMP6s and Floxed-ChR2(H134R)-EYFP mice crossed with hGFAP-CreERT2 mice (hGFAP-GCaMP6s and hGFAP-ChR2) were used in this study.
Wild animals	No wild animals were used in this study.
Field-collected samples	No field-collected samples were used in this study.
Ethics oversight	All experimental procedures were approved by the Seoul National University Institutional Animal Care and Use Committee and were conducted in accordance with Guide for the Care and Use of Laboratory Animals.

Note that full information on the approval of the study protocol must also be provided in the manuscript.
